# The superconductivity of Sr_2_RuO_4_ under *c*-axis uniaxial stress

**DOI:** 10.1038/s41467-022-32177-4

**Published:** 2022-08-06

**Authors:** Fabian Jerzembeck, Henrik S. Røising, Alexander Steppke, Helge Rosner, Dmitry A. Sokolov, Naoki Kikugawa, Thomas Scaffidi, Steven H. Simon, Andrew P. Mackenzie, Clifford W. Hicks

**Affiliations:** 1 Max Planck Institute for Chemical Physics of Solids, Nöthnitzer Str 40, 01187 Dresden, Germany; 2 Nordita, KTH Royal Institute of Technology and Stockholm University, Hannes Alfvéns väg 12, SE-106 91 Stockholm, Sweden; 3 National Institute for Materials Science, Tsukuba, 305-0003 Japan; 4 Department of Physics, University of Toronto, Toronto, ON M5S 1A7 Canada; 5 Department of Physics and Astronomy, University of California, Irvine, CA 92697 USA; 6 Rudolf Peierls Center for Theoretical Physics, Oxford, OX1 3PU UK; 7 Scottish Universities Physics Alliance, School of Physics and Astronomy, University of St. Andrews, St. Andrews, KY16 9SS UK; 8 School of Physics and Astronomy, University of Birmingham, Birmingham, B15 2TT UK

**Keywords:** Superconducting properties and materials, Electronic properties and materials

## Abstract

Applying in-plane uniaxial pressure to strongly correlated low-dimensional systems has been shown to tune the electronic structure dramatically. For example, the unconventional superconductor Sr_2_RuO_4_ can be tuned through a single Van Hove point, resulting in strong enhancement of both *T*_c_ and *H*_c2_. Out-of-plane (*c* axis) uniaxial pressure is expected to tune the quasi-two-dimensional structure even more strongly, by pushing it towards two Van Hove points simultaneously. Here, we achieve a record uniaxial stress of 3.2 GPa along the *c* axis of Sr_2_RuO_4_. *H*_c2_ increases, as expected for increasing density of states, but unexpectedly *T*_c_ falls. As a first attempt to explain this result, we present three-dimensional calculations in the weak interaction limit. We find that within the weak-coupling framework there is no single order parameter that can account for the contrasting effects of in-plane versus *c*-axis uniaxial stress, which makes this new result a strong constraint on theories of the superconductivity of Sr_2_RuO_4_.

## Abstract (web summary)

In the superconductor Sr2RuO4, in-plane strain is known to enhance both the superconducting transition temperature Tc and upper critical field Hc2, but the effect of out-of-plane strain has not been studied. Here, the authors find that Hc2 is enhanced under out-of-plane strain, but Tc unexpectedly decreases.

## Introduction

Sr_2_RuO_4_ is a famous exemplar of unconventional superconductivity, due to the quality of the available samples and the precision of knowledge about its normal state, and because the origin of its superconductivity remains unexplained in spite of strenuous effort^1–4^. No proposed order parameter is able straightforwardly to account for all the existing experimental observations. The greatest conundrum is posed by evidence that the order parameter combines even parity^5–8^ with time reversal symmetry breaking^9–11^. This combination of properties implies, if there is no fine tuning, that the superconducting order parameter is *d*_*xz*_ ± *id*_*yz*_^12^. Under conventional understanding, this is not expected because the horizontal line node at *k*_*z*_ = 0 implies interlayer pairing, while the electronic structure of Sr_2_RuO_4_ is highly two-dimensional^13,14^.

This puzzle has led to substantial theoretical activity. Two recent proposals are *s* ± *id*^15,16^ and *d* ± *ig*^17,18^ order parameters, which require tuning to obtain *T*_TRSB_ ≈ *T*_c_ (where *T*_TRSB_ is the time reversal symmetry breaking temperature), but avoid horizontal line nodes. A mixed-parity state^19^ and superconductivity that breaks time reversal symmetry only in the vicinity of extended defects^20^ have been proposed to account for the absence of a resolvable heat capacity anomaly at *T*_TRSB_^21^. Interorbital pairing through Hund’s coupling is also under discussion^22–25^; with some tuning of parameters this mechanism could yield *d*_*xz*_ ± *id*_*yz*_ order. Quasiparticle interference data, on the other hand, give evidence for a ${d}_{{x}^{2}-{y}^{2}}$-like gap, and a recent junction experiment shows time-reversal invariance^26,27^.

**Fig. 1:**
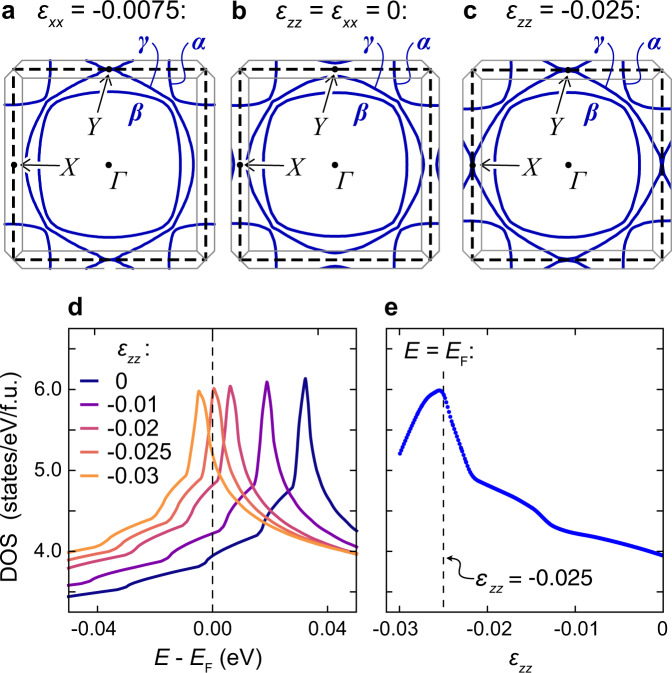
Electronic structure calculations of strained Sr_2_RuO_4_. **a**–**c** Cross-sections at *k*_*z*_ = 0 of calculated Fermi surfaces of Sr_2_RuO_4_ at the indicated strains. The heavy dashed lines indicate the zone of the RuO_2_ sheet, and the thin gray lines the 3D zone of Sr_2_RuO_4_. *X* and *Y* label high-symmetry points of the RuO_2_ zone. (To simplify discussion, we refer throughout this paper to the *X* and *Y* points defined in the 2D zone, rather than the points of the full 3D zone). **a** The electron-to-open Lifshitz transition induced by in-plane strain, from Ref. 29. It occurs at *ε*_*xx*_ = −0.0075 in the calculation, and *ε*_*xx*_ = −0.0044 experimentally^28^. **b** Unstressed Sr_2_RuO_4_. **c** The electron-to-hole Lifshitz transition under *c*-axis compression. **d** Calculated Fermi-level DOS against energy for a series of strains *ε*_*zz*_. **e** Calculated Fermi-level DOS against *ε*_*zz*_.

Uniaxial stress has become an important probe of the superconductivity of Sr_2_RuO_4_. When stress is applied along the [100] direction, the largest Fermi surface sheet (the *γ* sheet— see Fig. 1) distorts anisotropically, and undergoes a Lifshitz transition from an electron-like to an open geometry at −0.75 GPa (where negative values denote compression)^28^. The effect on the superconductivity is profound: *T*_c_ increases from 1.5 K in unstressed Sr_2_RuO_4_ to 3.5 K, while the *c*-axis upper critical field *H*_c2_ increases by a factor of twenty^29^. This very strong enhancement is qualitatively consistent with, for example, a ${d}_{{x}^{2}-{y}^{2}}$ order parameter. Under compression along the [100] direction, the Lifshitz transition occurs at approximately (*k*_*x*_, *k*_*y*_) = (0, ± *π*/*a*), which we label the *Y* point. As the transition occurs, the Fermi velocity at the *Y* point falls to nearly zero, which in general is expected to increase *T*_c_ and *H*_c2_ of order parameters such as ${d}_{{x}^{2}-{y}^{2}}$ in which the gap is large at the *Y* point. The data under [100] uniaxial stress argue against, for example, a *d*_*xy*_ order parameter.

Naively, then, *T*_c_ and *H*_c2_ might be expected to rise even further under compression along the *c* axis. *c*-axis compression raises the energy of the *d*_*xz*_ and *d*_*yz*_ bands relative to the *d*_*xy*_ band, and the resulting transfer of carriers expands the *γ* sheet, pushing it towards a Lifshitz transition from an electron-like to a hole-like geometry^30^. This transition occurs at both the *X* and *Y* points — see Fig. 1c — so the increase in the Fermi-level density of states (DOS) as it is approached is expected to be larger than for the electron-to-open Lifshitz transition induced by in-plane stress. Under *a*-axis compression *T*_c_ increases strongly well before the Lifshitz transition is reached, and so generically we expect this to occur for *c*-axis stress, too. The weak-coupling renormalization group study of Ref. 31 and functional renormalization group study of Ref. 32 both predict a rapid increase in *T*_c_ with approach to the electron-to-hole Lifshitz transition.

The electron-to-hole transition has been approached, and crossed, in thin films through epitaxial strain, and in bulk crystals by substitution of La for Sr^33–35^, but in both cases the superconductivity was suppressed by disorder. Here, we apply up to 3.2 GPa along the *c* axis of Sr_2_RuO_4_. This is a record uniaxial stress for bulk Sr_2_RuO_4_, and was achieved by sculpting samples with a focused ion beam to concentrate stress. *H*_c2_ increases, as expected from the increasing density of states. However, unexpectedly, *T*_c_ decreases. In other words, approaching the Lifshitz transition at either the *X* or *Y* point dramatically enhances *T*_c_, while approaching both suppresses *T*_c_. This is a major surprise. In a first attempt to address this issue we present calculations in the limit of weak coupling, that take into account the three-dimensional structure of the Fermi surfaces. Although these show that *c*-axis compression reduces the transition temperatures of certain order parameters, no order parameter could be identified for which the effects of both out-of-plane and in-plane pressure were captured. Our experimental finding therefore consitutes a major new constraint on theories of the superconductivity of Sr_2_RuO_4_.

## Results

### Electronic structure calculations

We start with density functional theory (DFT) calculations of Sr_2_RuO_4_ under *c*-axis compression, as a guide to the likely effects of *c*-axis strain on the electronic structure. Figure 1 shows our results. Panel a shows the Fermi surfaces under 0.75% compression along the *a* axis, panel b those of the unstrained lattice, and panel c those under 2.5% compression along the *c* axis. The calculations are done under conditions of uniaxial stress, meaning that the transverse strains are the longitudinal strain times the relevant Poisson’s ratios for Sr_2_RuO_4_. DFT calculations reproduce well the changes under [100] uniaxial stress observed in ARPES measurements^36^. Technical details of the calculation are provided in the Methods section.

The calculations predict that the electron-to-hole transition will occur at *ε*_*zz*_ = −0.025. Under *a*-axis compression, these calculations predict that the electron-to-open transition occurs at *ε*_*xx*_ = −0.0075, whereas it was observed experimentally to occur at *ε*_*xx*_ = −0.0044^28^, so this *c*-axis prediction might similarly overestimate the level of compression required. The uncertainty arises from the fact that the distance to the Lifshitz transition is sensitive to meV-level energy shifts, likely driven by many-body renormalisation^28^. Low-temperature ultrasound data give a *c*-axis Young’s modulus of 219 GPa^37^, so *ε*_*zz*_ = −0.025 corresponds to *σ*_*zz*_ ≈ −5.5 GPa. Separately, we note also that while *k*_*z*_ warping increases on all the Fermi sheets, as expected for *c*-axis compression, the *β* sheet has the strongest *k*_*z*_ warping both at *ε*_*zz*_ = 0 and at the Lifshitz transition; see the Methods section for an illustration.

### Experimental results

Four samples were measured. For good stress homogeneity, samples should be elongated along the stress axis, which is a challenge for the *c* axis because the cleave plane of Sr_2_RuO_4_ is the *ab* plane. A plasma focused ion beam, in which material is milled using a beam of Xe ions, was therefore used to shape the samples. Sample 1 was prepared with a uniform cross section, and a large enough stress, *σ*_*zz*_ = −0.84 GPa, was achieved to observe a clear change in *T*_c_. To go further, the other samples were all sculpted into dumbell shapes, with the wide ends providing large surfaces for coupling force into the sample. FIB microstructuring has been used to achieve large *c*-axis stress in CaFe_2_As_2_^38^, but here we needed to retain sufficient sample volume for high-precision magnetic susceptibility measurements. For measurement of *T*_c_ in the neck portion, two concentric coils of a few turns each were wound around the neck. Samples 1 and 4 also had electrical contacts, for measurement of the *c*-axis resistivity *ρ*_*zz*_. Photographs of samples 2 and 4 are shown in the Methods section.

Sample 4 was measured in apparatus that incorporated a sensor of the force applied to the sample^39^, from which the stress in the sample could be accurately determined. Samples 1–3 were mounted into apparatus that had a sensor only of the displacement applied to the sample, which is an imperfect measure of the sample strain because the measured displacement includes deformation in the epoxy that holds the sample. Therefore, a displacement-to-stress conversion was applied to samples 1–3 to bring the rate of change of *T*_c_ over the stress range 0.92 < *σ*_*zz*_ < − 0.20 GPa into agreement with that of sample 4. In other words, we impose on our data an assumption that the initial rate of decrease in *T*_c_ is the same in all the samples, which is reasonable because their zero-stress *T*_c_’s are very similar: all are between 1.45 and 1.50 K.

**Fig. 2:**
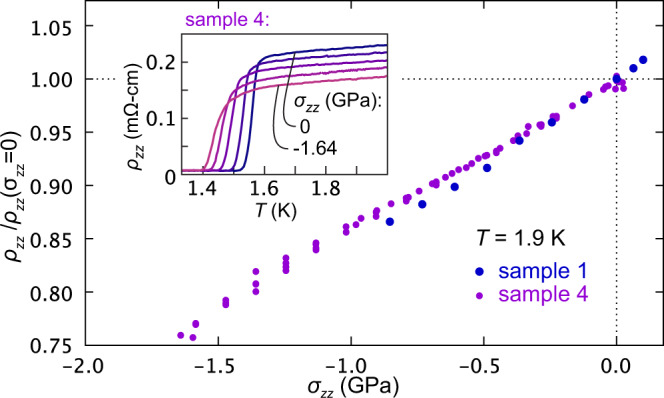
Electrical transport under c-axis stress. Main panel: *c*-axis resistivity *ρ*_*zz*_ versus stress *σ*_*zz*_ at 1.9 K, normalized by its *σ*_*zz*_ = 0 value. Note that the stress scale of sample 1 is adjusted so that *dT*_*c*_/*dσ*_*zz*_ as measured through the Meissner effect matches that from sample 4 over the range − 0.92 < *σ*_*zz*_ < − 0.2 GPa. At *σ*_*zz*_ = 0, *ρ*_*zz*_(1.9K) of samples 1 and 4 is 0.278 and 0.228 mΩ-cm, respectively. Inset: *ρ*_*zz*_ versus temperature for sample 4 at 0, − 0.37, − 0.85, − 1.25, and − 1.64 GPa.

We begin by showing resistivity data, in Fig. 2. The plotted resistivities are corrected for the expected stress-induced change in sample geometry (reduced length and increased width), using the low-temperature elastic moduli reported in Ref. 37, and making the assumption that stress and strain are linear over this entire range. At zero stress the resistivity of sample 4 shows a sharp transition into the superconducting state at 1.55 K. This sharpness, and the fact that it only slightly exceeds the transition temperature seen in susceptibility, indicate high sample quality. With compression, *T*_c_ decreases. The normal-state resistivity also decreases, following the general expectations that *c*-axis compression should increase *k*_*z*_ dispersion.

We find elastoresistivities (1/*ρ*_*zz*_)*dρ*_*zz*_/*dε*_*zz*_, obtained from linear fits over the range − 0.5 < *σ*_*zz*_ < 0 GPa, of 37 and 32 for samples 1 and 4, respectively. Sample 4 was compressed to −1.7 GPa, and its resistivity does not show any major deviation from linearity over this range. The scatter in the data at strong compression may be a consequence of cracking in the electrical contacts— we show below that the sample deformation was almost certainly elastic.

**Fig. 3:**
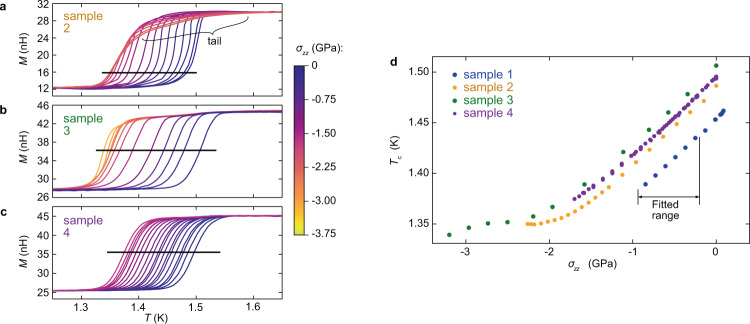
*T*_c_ under uniaxial stress. **a**–**c** Mutual inductance *M* of the sense coils versus temperature for samples 2–4, at various applied stresses *σ*_*zz*_. The lines indicate the selected thresholds for determination of *T*_c_. For sample 2, under large ∣*σ*_*zz*_∣ a tail appears on the transitions, which we attribute to in-plane stress, and a low threshold is chosen to avoid this tail. **d** *T*_c_ versus stress for all the samples. The stress scales for samples 1–3 are scaled to bring *dT*_c_/*dσ*_*zz*_ into agreement with that of sample 4 over the stress range labeled “fitted range”, − 0.92 to − 0.20 GPa.

We now show the effects of *c*-axis compression on magnetic susceptibility. Figure 3a–c shows the transitions of samples 2–4 in susceptibility; the data shown are the mutual inductance *M* of the sense coils versus temperature. To check that sample deformation remained elastic, we repeatedly cycled the stress to confirm that the form of the *M*(*T*) curves remained unchanged; see the Methods section for examples. For samples 3 and 4, the transition remained narrow as stress was applied, indicating high stress homogeneity. For sample 2, there was a tail on the high-temperature side of the transition, that was stronger at higher compressions. We attribute it to in-plane strain, possibly originating in the fact that sample 2 was not as well aligned as samples 3 and 4. A similar, though weaker, tail is also visible for sample 3.

We note that the width of the transitions in Fig. 3a–c — ≈ 50 mK — will be a consequence of defects and/or an internal distribution in the in-plane strain. Although there will also be inhomogeneity in *ε*_*zz*_, this is not the driver of the transition width: the distribution would have to have a width of ~1 GPa, which is not plausible.

Figure 3 (d) shows *T*_c_ versus stress for all the samples. *T*_c_ is taken as the temperature where *M* crosses a threshold. For samples 1, 3, and 4, we select a threshold at ≈ 50% of the height of the transition, and for sample 2, 20%, in order to minimize the influence from the high-temperature tail. *T*_c_ is seen to decrease almost linearly out to *σ*_*zz*_ ≈ − 1.8 GPa. For sample 4 (to which, as described above, the other samples are referenced), *dT*_c_/*dσ*_*zz*_ in the limit *σ*_*zz*_ → 0 is 76 ± 5 mK/GPa. The error is 6%: we estimate a 5% error on the calibration of the force sensor of the cell, and a 3% error on the cross-sectional area of the sample (155 × 106 *μ*m^2^).

At *σ*_*zz*_ ≲ − 1.8 GPa, the stress dependence of *T*_c_ flattens markedly. In sample 3, *T*_c_ resumes its decrease for *σ*_*zz*_ < − 3 GPa. We show in the Methods section that both the flattening and this further decrease reproduce when the stress is cycled, which, in combination with the narrowness of the transitions, shows that this behavior is intrinsic, not an artefact of any drift or non-elastic deformation in the system.

**Fig. 4:**
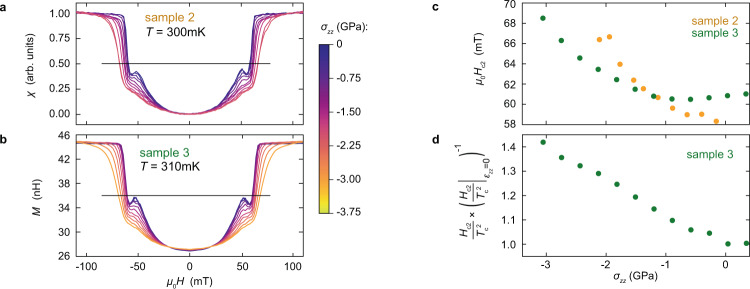
*H*_c2_ under c-axis stress. **a**, **b** Susceptibility versus applied field ∥*c* for samples 2 and 3, at fixed temperatures of 300 mK and 310 mK. For sample 3, the raw data are plotted. For sample 2, the signal magnitude shifted from run to run, probably due to slipping of the coils against the sample, so data are normalized by the readings at 0 and 100 mT. The horizontal lines indicate the thresholds for determination of *H*_c2_. **c** *H*_c2_(*T* ≈ 0.3K) versus stress for samples 2 and 3. **d** ${H}_{{{{{{{{\rm{c2}}}}}}}}}/{T}_{{{{{{{{\rm{c}}}}}}}}}^{2}$, normalized by its zero-stress value, versus stress for sample 3.

Figure 4 shows measurements of the *c*-axis upper critical field. *M*(*H*) for samples 2 and 3 at constant temperature *T* ≈ 0.3 K is shown in panels a and b. In Fig. 4c, we plot *H*_c2_ versus stress, taking *H*_c2_ as the fields at which *M* crosses the thresholds indicated in panels a–b. *H*_*c*2_ increases as stress is applied, as generally expected when the density of states increases. The increase is faster for sample 2 than sample 3, which may be an artefact of the tail on the transition for sample 2.

The quantity ${H}_{{{{{{{{\rm{c2}}}}}}}}}/{T}_{{{{{{{{\rm{c}}}}}}}}}^{2}$ is particularly informative: if pairing strength were modified without changing the gap structure, *H*_c2_ would be proportional to ${T}_{{{{{{{{\rm{c}}}}}}}}}^{2}$. As shown in Fig. 4d, we observe ${H}_{{{{{{{{\rm{c2}}}}}}}}}/{T}_{{{{{{{{\rm{c}}}}}}}}}^{2}$ to increase by 40% by *σ*_*zz*_ = − 3.0 GPa. Slower quasiparticles are less strongly affected by magnetic field, so what this result means is that the Fermi velocity decreases on portions of the Fermi surface where the gap is large.

We conclude this section with a note on the peak effect — the small maximum in the susceptibility just below *H*_c2_, visible in Fig. 4 (a, b). The peak effect occurs when there is a range of temperature below *T*_c_ where vortex motion is uncorrelated, allowing individual vortices to find deeper pinning sites^40^. The peak is suppressed by *c*-axis compression, and it is suppressed downward rather than by being smeared horizontally along the *H* axis, which means that this suppression is not an artefact of a spread in *H*_c2_ due to strain inhomogeneity. It could indicate stronger pinning, due to the reduction in the coherence length.

### Weak-coupling calculations

As explained in the introduction, there is an apparent contradiction between the increase of *T*_c_ under *a*-axis strain reported in previous work (which suggests anti-nodes at the *X* and *Y* points) and the decrease of *T*_c_ under *c*-axis pressure (which suggests nodes at the *X* and *Y* points). To see if this puzzle has a straightforward solution, we perform weak-coupling calculations for repulsive Hubbard models, as developed in Refs. 41–50. To capture possible changes in the 3D gap structure, we employ three-dimensional Fermi surfaces^51^. These are described by a three-band (4*d* *xy*, *xz*, and *yz*) tight-binding model. The hopping integrals are derived from the Ru-centred Wannier functions obtained in the DFT calculation presented above. Our tight-binding model takes the form

1 $${H}_{0}=\mathop{\sum}\limits_{{{{{{{{\bf{k}}}}}}}},s}{{{{{{{{\boldsymbol{\psi }}}}}}}}}_{s}^{{{{\dagger}}} }({{{{{{{\bf{k}}}}}}}}){{{{{{{{\mathcal{H}}}}}}}}}_{s}({{{{{{{\bf{k}}}}}}}}){{{{{{{{\boldsymbol{\psi }}}}}}}}}_{s}({{{{{{{\bf{k}}}}}}}}).$$

${{{{{{{{\boldsymbol{\psi }}}}}}}}}_{s}({{{{{{{\bf{k}}}}}}}})={[{c}_{xz,s}({{{{{{{\bf{k}}}}}}}}),{c}_{yz,s}({{{{{{{\bf{k}}}}}}}}),{c}_{xy,\bar{s}}({{{{{{{\bf{k}}}}}}}})]}^{T}$, and ${{{{{{{{\mathcal{H}}}}}}}}}_{s}({{{{{{{\bf{k}}}}}}}})$ incorporates spin-orbit coupling, inter-orbital and intra-orbital terms. The complete set of tight-binding parameters retained here is given in the Methods section.

**Fig. 5:**
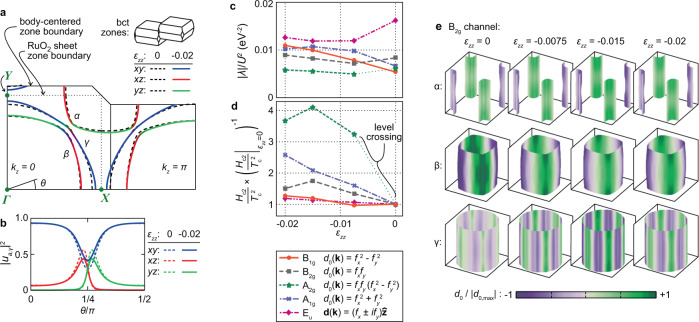
Weak-coupling calculations of Sr_2_RuO_4_ under c-axis strain. **a** Cuts through the tight-binding Fermi surfaces employed in our weak-coupling calculations, at *ε*_*zz*_ = 0 and − 0.02. The surfaces at *ε*_*zz*_ = − 0.02 are colored by orbital content. The solid lines show the Brillouin zone boundaries of the body-centred tetragonal unit cell of Sr_2_RuO_4_, and the dotted line of the 2D zone of the RuO_2_ sheets. **b** Orbital weights on the *γ* sheet at *k*_*z*_ = 0 in this model. **c** Leading eigenvalues as a function of *ε*_*zz*_ for *J*/*U* = 0.15, and **d** ${H}_{{{{{{{{\rm{c2}}}}}}}}}(T\to 0)/{T}_{{{{{{{{\rm{c}}}}}}}}}^{2}$, normalised by its value at zero strain, of the leading eigenstate versus *ε*_*zz*_ in each channel. In the legend, *f*_*i*_ is any function that transforms as $\sin {k}_{i}$, *d*_0_(**k**) is the gap function for the even-parity irreducible representations, and **d**(**k**) the *d*-vector for *E*_*u*_. In the *A*_2*g*_ channel, there is a level crossing between *ε*_*zz*_ = 0 and − 0.0075, that results an anomalously strong increase in ${H}_{{{{{{{{\rm{c2}}}}}}}}}/{T}_{{{{{{{{\rm{c}}}}}}}}}^{2}$. **e** Gap structure in the *B*_2g_ channel versus *ε*_*zz*_, on the *α*, *β*, and *γ* Fermi sheets.

In Fig. 5a, we show the tight-binding Fermi surfaces at *ε*_*zz*_ = 0 and − 0.02. In Fig. 5b, we show the orbital weight on the *γ* sheet at *k*_*z*_ = 0. As the *γ* sheet expands, the orbital mixing around its avoided crossings with the *β* sheet is reduced, and it becomes more dominated by *xy* orbital weight.

To *H*_0_ we add on-site Coulomb terms projected onto the *t*_2*g*_ orbitals^52^ (Methods Eq. (8)) and study the solutions to the linearized gap equation in the weak-coupling limit *U*/*t* ≪ 1, where *U* is the intraorbital Coulomb repulsion and *t* is the leading tight-binding term. We take the interorbital on-site Coulomb repulsion to be ${U}^{\prime}=U-2J$, where *J* is the Hund’s coupling, and the pair-hopping Hund’s interaction ${J}^{\prime}$ to be equal to the spin-exchange Hund’s interaction *J*. Under these assumptions, the remaining free parameter is *J*/*U*. We take *J*/*U* = 0.15, which is close to the value *J*/*U* = 0.17 found in Refs. 53,54. The linearized gap equation reads

2 $$\mathop{\sum}\limits_{\nu }{\int}_{{S}_{\nu }}\frac{{{{{{{{\rm{d}}}}}}}}{{{{{{{{\bf{k}}}}}}}}}_{\nu }}{|{S}_{\nu }|}\bar{{{\Gamma }}}({{{{{{{{\bf{k}}}}}}}}}_{\mu },{{{{{{{{\bf{k}}}}}}}}}_{\nu })\varphi ({{{{{{{{\bf{k}}}}}}}}}_{\nu })=\lambda \varphi ({{{{{{{{\bf{k}}}}}}}}}_{\mu }),$$

where *μ* and *ν* are band indices, ∣*S*_*ν*_∣ is the area of Fermi surface sheet *ν*, and $\bar{{{\Gamma }}}$ is the two-particle interaction vertex calculated consistently to order ${{{{{{{\mathcal{O}}}}}}}}({U}^{2}/{t}^{2})$. Solutions to Eq. (2) with *λ* < 0 signal the onset of superconductivity, at the critical temperature ${T}_{c} \sim W\exp (-1/|\lambda|)$, where *W* is the bandwidth.

In a pseudo-spin basis each eigenvector *φ* belongs to one of the ten irreducible representations of the crystal point group *D*_4*h*_^48,55^. We calculate the leading eigenvalues in four even-parity channels, *B*_1*g*_, *B*_2*g*_, *A*_1*g*_, and *A*_2*g*_ — see the legend of Fig. 5c–d. The *E*_*g*_ channel — *d*_*xz*_ ± *id*_*yz*_ — has been found to be strongly disfavored in weak-coupling calculations^51^, and so is not considered here.

A subset of the present authors have found, in a previous weak-coupling calculation, that the odd-parity order parameters track each other closely as *J*/*U* is varied, with a splitting that is small compared with that between the even-parity orders, and between the odd- and even-parity orders^51^. Ref. 56, likewise, finds the splitting between the odd-parity orders to be small. For this reason, we calculate only one odd-parity channel, *E*_*u*_ (*p*_*x*_ ± *ip*_*y*_), and its behaviour can safely be taken to represent the qualitative behaviour of odd-parity order.

The leading eigenvalues in each channel as a function of *ε*_*zz*_ are shown in Fig. 5c. Although, as in Ref. 51, odd-parity order is found to be favored, calculations in the random phase approximation at similar *J*/*U* tend to favor even-parity order^15,56^. A tendency towards odd-parity order appears to be a feature of calculations in the weak-coupling limit. We note also that the ordering of the channels differs from what was found in Ref. 51, due to a different tight-binding parametrisation. The ordering is sensitive to the parametrisation, and so we focus discussion here on trends with applied strain.

The weak-coupling results show a dichotomy in the strain dependence of *T*_c_: *T*_c_ in the channels that have symmetry-imposed nodes at the *X* and *Y* points (*E*_*u*_, *A*_2*g*_, and *B*_2*g*_) decreases with initial *c*-axis compression. These nodes coincide with the regions of highest local density of states, and this result is an indication that order parameters in these channels are less able to take advantage of the increase in Fermi-level density of states induced by *c*-axis compression. However, under stronger compression *T*_c_ increases modestly in all channels.

In the weak-coupling calculations of Ref. 29, the contrast in the response to *a*-axis uniaxial stress between order parameters with and without nodes at the *X* and *Y* points was found to be stronger in ${H}_{{{{{{{{\rm{c2}}}}}}}}}/{T}_{{{{{{{{\rm{c}}}}}}}}}^{2}$ than *T*_c_, and so we also calculate ${H}_{{{{{{{{\rm{c2}}}}}}}}}/{T}_{{{{{{{{\rm{c}}}}}}}}}^{2}$, following the procedure in Ref. 29. Results are shown in Fig. 5d. We find that changes in ${H}_{{{{{{{{\rm{c2}}}}}}}}}/{T}_{{{{{{{{\rm{c}}}}}}}}}^{2}$ correlate closely with shifts in the gap weight onto the *γ* sheet, which has the lowest Fermi velocity. For example, gap weight in the *B*_2*g*_ channel, shown in Fig. 5e, shifts from the *β* to the *γ* sheet as stress is initially applied, and ${H}_{{{{{{{{\rm{c2}}}}}}}}}/{T}_{{{{{{{{\rm{c}}}}}}}}}^{2}$ correspondingly increases. At strong compression, gap weight shifts back to the *β* sheet, and ${H}_{{{{{{{{\rm{c2}}}}}}}}}/{T}_{{{{{{{{\rm{c}}}}}}}}}^{2}$ decreases. This occurs because as the *γ* sheet expands it comes closer to its copies in adjacent zones, which disfavours a large gap on this sheet because in the *B*_2*g*_ channel the gap changes sign across the zone boundary. Among the even-parity channels, for *ε*_*zz*_ < − 0.015${H}_{{{{{{{{\rm{c2}}}}}}}}}/{T}_{{{{{{{{\rm{c}}}}}}}}}^{2}$ increases for those without nodes along the Γ-*X* and Γ-*Y* lines, and decreases for those with. The complete set of calculated gap structures is shown in the Methods section.

We conclude this section by noting that although a *k*_*z*_ dependence of the gap structure is seen in all channels, we do not find dramatic stress-induced changes in the *k*_*z*_ dependence in any channel. Separately, in the *A*_2*g*_ channel there is a level crossing between *ε*_*zz*_ = 0 and −0.0075. We plot only the leading eigenvalues in Fig. 5; this level crossing causes a large change in the leading gap structure and an anomalously large increase in ${H}_{{{{{{{{\rm{c2}}}}}}}}}/{T}_{{{{{{{{\rm{c}}}}}}}}}^{2}$.

## Discussion

The unexpected decrease of *T*_c_ as two Van Hove points in *k*-space are approached under *c*-axis compression is the key experimental result that we report. It might provide a vital clue about the nature of the superconducting state in Sr_2_RuO_4_, because it is so different to the response to in-plane, *a*-axis pressure. The DFT calculations indicate that our largest achieved stress, −3.2 GPa, is around 60% of the way to the Lifshitz transition, and if the calculations overestimate the compression required to reach the Lifshitz transition, as they did for in-plane stress, we might have come even closer. The contrast between the effects of *a*- and *c*-axis stress is unmistakable: 60% of the way to the electron-to-open Lifshitz transition under *a*-axis stress, *T*_c_ is 0.7 K higher than in the unstressed sample^28^.

The response of *T*_c_ under *c*-axis compression allows resolution of the stress dependence of *T*_c_ into components through comparison with the the effect of hydrostatic compression, which also suppresses *T*_c_. We obtain the coefficients *α* and *β* in the expression

3 $${T}_{{{{{{{{\rm{c}}}}}}}}}={T}_{{{{{{{{\rm{c}}}}}}}},0}+\alpha \times \frac{{{\Delta }}V}{V}+\beta \times \left({\varepsilon }_{zz}-\frac{{\varepsilon }_{xx}+{\varepsilon }_{yy}}{2}\right),$$

where Δ*V*/*V* = *ε*_*xx*_ + *ε*_*yy*_ + *ε*_*zz*_ is the fractional volume change of the unit cell, and *ε*_*zz*_ − (*ε*_*xx*_ + *ε*_*yy*_)/2 is a volume-preserving tetragonal distortion. Refs. 12,57,58 report *dT*_c_/*dσ*_hydro_ = 0.22 ± 0.02, 0.24 ± 0.02, and 0.21 ± 0.03 K/GPa; we take *dT*_c_/*dσ*_hydro_ = 0.23 ± 0.01 K/GPa. Employing the low-temperature elastic moduli from Ref. 37 to convert stress to strain, we find *α* = 34.8 ± 1.6 K and *β* = − 2.2 ± 1.2 K. (Under hydrostatic stress, *σ*_*zz*_/*ε*_*zz*_ = 396 GPa and *ε*_*xx*_ = 0.814*ε*_*zz*_.) The small value of *β* means that a volume-preserving reduction in the lattice parameter ratio *c*/*a* would have little effect on *T*_c_: the increase in density of states by approaching the electron-to-hole Lifshitz transition is balanced, somehow, by weakening of the pairing interaction. The challenge for theory is to understand why that weakening takes place.

In the three-dimensional weak-coupling calculations presented here, it is the *A*_2*g*_ and *B*_2*g*_ channels, both of which have nodes along the Γ-*X* and Γ-*Y* lines, that best match observations. Due to differences between the actual and tight-binding electronic structure the *ε*_*zz*_ = 0 point in the calculations should not be considered too literally as equivalent to *ε*_*zz*_ = 0 in reality, and the key point is that it is only in the *A*_2*g*_ and *B*_2*g*_ channels that *T*_*c*_ is found to decrease and ${H}_{{{{{{{{\rm{c2}}}}}}}}}/{T}_{{{{{{{{\rm{c}}}}}}}}}^{2}$ to increase over some range of strain. However, as we have noted, *A*_2*g*_ and *B*_2*g*_ order parameters do not appear to be consistent with data under *a*-axis stress.

In other words, weak-coupling calculations do not explain the contrasting responses to *a*- versus *c*-axis stress, and this provides an opportunity: models of pairing in Sr_2_RuO_4_ should be tested against this feature, for it might provide substantial resolving power between different models. There may, for example, be stress-driven changes in the interactions that drive superconductivity, though to attempt to calculate this is beyond the scope of this paper.

We highlight two other possible explanations. One is interorbital pairing^22–25^. The superconducting energy scale is too weak to induce substantial band mixing, and so these models depend on the proximity of the *γ* and *β* sheets, and the resulting mixing of *xy* and *xz*/*yz* orbital weight over substantial sections of Fermi surface^23^. We have noted that *c*-axis compression reduces this mixing, by pushing the *γ* and *β* sheets apart, which could then suppress *T*_c_^59^. In contrast, under in-plane uniaxial compression these sheets are pushed closer together along one direction and further apart along the other^36^.

The other is three-dimensional effects. Another feature of the electronic structure that varies oppositely under *a*- versus *c*-axis compression is the interlayer coupling. Under *a*-axis compression, the RuO_2_ sheets are pushed further apart, and under *c*-axis compression, closer together. For example, an increase in warping of the Fermi surfaces along *k*_*z*_ under *c*-axis compression could reduce the quality of nesting and so weaken spin fluctuations in Sr_2_RuO_4_, and the weak-coupling calculations here might not have fully captured the effect on the superconductivity.

In summary, we have demonstrated methods to apply uniaxial stress of multiple GPa along the interlayer axis of layered materials in samples large enough to permit high-precision magnetic susceptibility measurements. Under such a compression, we find that *T*_c_ decreases even though the Fermi-level DOS increases, in striking contrast to the effect of in-plane uniaxial stress. Weak-coupling calculations do not provide a clear answer to this puzzle, which makes it important for models of superconductivity in Sr_2_RuO_4_ to be tested against application of both types of stress. At a more general level, our findings motivate the use of out-of-plane stress as a powerful tool for investigation of other low dimensional strongly correlated system in which the strength of the interlayer coupling is suspected of playing an important role in their electronic properties.

## Methods

### Density functional theory calculation

DFT structure calculations were performed using the full-potential local orbital FLPO^60,61^ version fplo 18.00-52 (http://www.fplo.de). For the exchange-correlation potential, the local density approximation applying the parametrizations of Perdew-Wang^62^ was chosen. Spin-orbit coupling was treated non-perturbatively by solving the four-component Kohn-Sham-Dirac equation^63^. To obtain precise band structure and Fermi surface information in the presence of a Van Hove singularity close to the Fermi level, the final calculations were carried out on a well-converged mesh of 343,000 *k* points. (70 × 70 × 70; 23,022 points in the in the irreducible wedge of the Brillouin zone). As a starting point, for the unstrained lattice structure the structural parameters at 15 K from Ref. 64 were used. Longitudinal strain *ε*_*zz*_ is taken as the independent variable, and *ε*_*xx*_ and *ε*_*yy*_ are set following the low-temperature Poisson’s ratio from Ref. 37, which is 0.223 for stress along the *c* axis. The apical oxygen position was relaxed independently at each strain, by minimising the force to below 1 meV/Å. However, the effect of relaxing this internal parameter is small in comparison with the effect of the stress-driven change in lattice parameters.

**Fig. 6:**
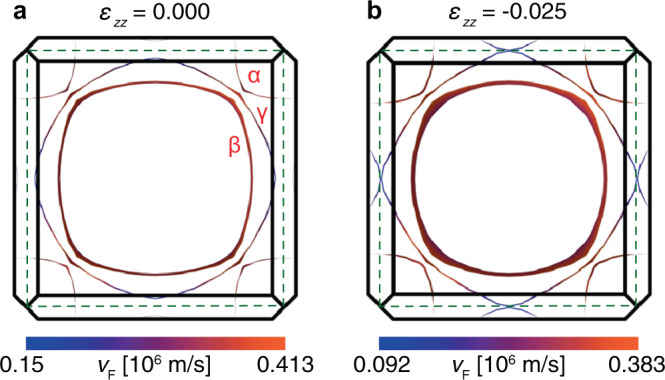
DFT Fermi surfaces under *c*-axis compression. Fermi surfaces of Sr_2_RuO_4_ projected along *k*_*z*_ under (**a**) zero stress and (**b**) *ε*_*zz*_ = − 0.025. The width of the lines indicates the warping of the Fermi surface along *k*_*z*_. The dashed green line is the 2D zone boundary of the RuO_2_ sheet.

The calculated Fermi surfaces of unstressed Sr_2_RuO_4_ and under interlayer compression, including the warping along *k*_*z*_ and the Fermi velocities, are shown in Fig. 6. The *β* sheet is the most strongly warped both at zero stress and at *ε*_*zz*_ = −0.025.

### Experimental details

Sr_2_RuO_4_ samples were grown using a floating-zone method^65,66^. The four samples here were taken from the same original rod, and from a portion that we verified to have high *T*_c_ and a low-density of Ru inclusions; our aim in taking multiple samples was to test reproducibility in sample preparation and mounting.

Uniaxial stress was applied using piezoelectric-driven apparatus^39,67^, and precision in sample mounting is important because Sr_2_RuO_4_ is much more sensitive to in-plane than *c*-axis uniaxial stress: *T*_c_ decreases by 0.13 K under a *c*-axis stress of *σ*_*zz*_ = − 3.0 GPa, but increases by 0.13 K under an in-plane uniaxial stress of only 0.2 GPa^29^. Applying *c*-axis pressure could generate in-plane stress through bending and/or sample inhomogeneity. In a previous experiment^68^, *c*-axis compression raised *T*_c_ and broadened the transition. However, the stress was applied at room temperature, where the elastic limit of Sr_2_RuO_4_ is low^39^, so these effects may have been a consequence of in-plane strain due to defects introduced by the applied stress.

**Fig. 7:**
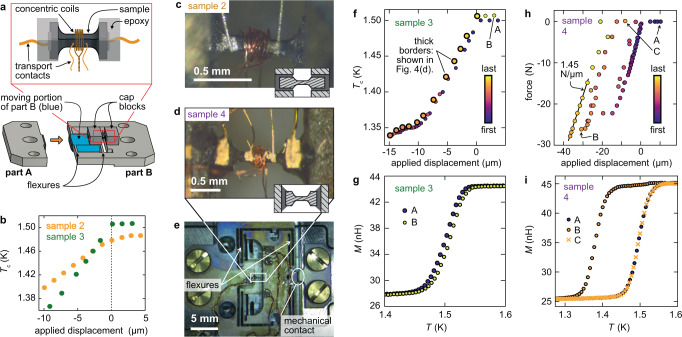
Sample setup. **a** Schematic of the sample configuration for samples 2 and 3. These samples were sculpted into a dumbell shape with a plasma focused ion beam. They were mounted with their ends embedded in epoxy. The cap blocks incorporate slots into which the sample fits, and the sample is compressed by bringing parts A and B into contact. **b** *T*_c_ versus applied displacement for samples 2 and 3. When parts A and B are brought into contact, force is applied to the sample, and *T*_c_ changes. **c**, **d** Photographs of samples 2 and 4. The graphics at the lower right of each panel are schematic cross sections: the end tabs of samples 2 were epoxied into slots, while sample 4 was sandwiched between two surfaces. **e** A photograph of the sample carrier for sample 4. **f** *T*_c_ of sample 3 versus applied displacement, with the data points coloured by the order in which they were taken. The sequence of data points with thick borders, during which ∣*σ*_*zz*_∣ was monotonically decreased, are those shown in Fig. 3d. **g** Sense coil mutual inductance *M*(*T*) at points A and B in panel (**f**). **h** Force versus displacement for sample 4. **i** *M*(*T*) at points A, B, and C in panel (**h**).

Samples 2–4 were mounted into two-part sample carriers; that for samples 2 and 3 is diagrammed in Fig. 7a. The purpose was to protect samples from inadvertent application of tensile stress. Samples are mounted across a gap between a fixed and a moving portion of part B of the carrier, and can be compressed, but not tensioned, by bringing part A into contact with part B. In Fig. 7b, we show *T*_c_ of samples 2 and 3 versus applied displacement, and the point where parts A and B come into contact and *T*_c_ starts changing is clearly visible. For sample 2 the point of contact is rounded on the scale of a few microns, due to roughness and/or misalignment of the contact faces, and in all figures below we exclude data points that we estimate to be affected by this rounding.

The samples were mounted with Stycast 2850. This epoxy layer constitutes a conformal layer that ensures even application of stress^67^. Photographs of samples 2 and 4 are shown in Fig. 7c and d. The carrier for sample 4, which has a different design to those used for samples 2 and 3, is shown in Fig. 7e. Where electrical contacts were made, Du-Pont 6838 silver paste annealed at 450^∘^ for typically 30 min was used. This is longer than usual, in order to penetrate a thin insulating layer deposited during the ion beam milling.

As noted above, samples 1–3 were mounted in apparatus that had a sensor only of the displacement applied to the sample, while for sample 4 there was also a force sensor. Displacement sensors are less reliable as sensors of the state of the sample, because they also pick up deformation of the epoxy that holds the sample. In Fig. 7f the complete set of measurements of *T*_c_ of sample 3, plotted against applied displacement, are shown. Data points are colored by the order in which they were collected. The data drifted leftward over time: stronger compression was needed to reach the same *T*_c_. However, the qualitative form of the curve — initial decrease in *T*_*c*_, then a flattening, and then further decrease — reproduced over multiple stress cycles, and in Fig. 7g it is shown that the form of the transition was the same before and after application of the strongest compression. (We attribute the small apparent shift in *T*_c_ to an artefact of inadvertent mechanical contact between the stress cell and inner vacuum can of the cryostat).

We therefore conclude that the sample deformed elastically and that it was the epoxy holding the sample that was compressed non-elastically; plastic deformation has previously been observed to broaden the superconducting transition^69^ of Sr_2_RuO_4_. In Fig. 3, in the main text, we show only the data taken after the epoxy was maximally compressed. Force versus displacement data for sample 4 are shown in Fig. 7h–i, and here it can be seen that there was very substantial non-elastic compression of the epoxy. As with sample 3, the shape of the superconducting transition in the Sr_2_RuO_4_ was the same before and after application of large stress. Over regions where the sample and epoxy deformed elastically, the combined spring constant was 1.45 N/*μ*m. The spring constant of the flexures in the carrier, on the other hand, is calculated to be ~0.03 N/*μ*m, meaning that almost all of the applied force was transferred to the sample.

### Calculated gap structure in other channels

**Fig. 8:**
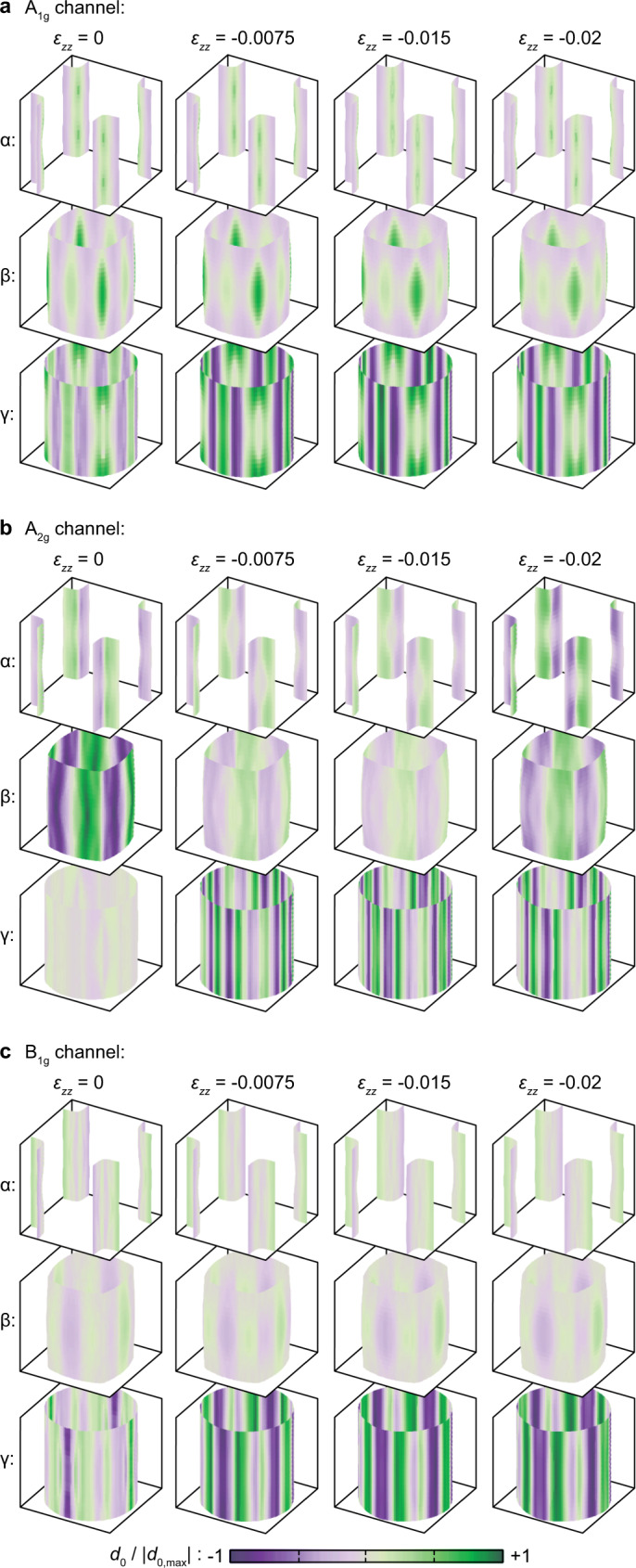
Additional gap structures. Gap structures at *J*/*U* = 0.15 for, from (**a**–**c**), the *A*_1*g*_, *A*_2*g*_, and *B*_1*g*_ channels, at the indicated strains. For each channel, the top, middle, and bottom rows show the gap on the *α*, *β*, and *γ* sheets, respectively.

In Fig. 8 the calculated gap structures in the *A*_1*g*_, *A*_2*g*_, and *B*_1*g*_ channels are shown. [The *B*_2*g*_ gap structures are shown in Fig. 5e.] *c*-axis compression favors large gaps on the *γ* sheet in all channels. In the *A*_2*g*_ channel, this shift occurs as a first-order change in gap structure between *ε*_*zz*_ = 0 and *ε*_*zz*_ = − 0.0075. At the largest compression reached, gap weight in the *A*_2*g*_ channel shifts back away from the *γ* sheet, as it does in the *B*_2*g*_ channel. This does not occur in the *A*_1*g*_ and *B*_1*g*_ channels.

### Details of the weak-coupling calculation

The tight-binding Hamiltonian from Eq. (1) takes the form

4 $${{{{{{{{\mathcal{H}}}}}}}}}_{s}({{{{{{{\bf{k}}}}}}}})=\left(\begin{array}{ccc}{\varepsilon }_{AA}({{{{{{{\bf{k}}}}}}}})&{\varepsilon }_{AB}({{{{{{{\bf{k}}}}}}}})-is{\eta }_{1}&+i{\eta }_{2}\\ {\varepsilon }_{BA}({{{{{{{\bf{k}}}}}}}})+is{\eta }_{1}&{\varepsilon }_{BB}({{{{{{{\bf{k}}}}}}}})&-s{\eta }_{2}\\ -i{\eta }_{2}&-s{\eta }_{2}&{\varepsilon }_{CC}({{{{{{{\bf{k}}}}}}}})\end{array}\right),$$

where we used the Ru orbital shorthand notation *A* = *xz*, *B* = *yz*, *C* = *xy*, and where $\bar{s}=-s$ (*s* being spin). In Eq. (4) the energies *ε*_*AB*_(**k**) account for intra-orbital (*A* = *B*) and inter-orbital (*A* ≠ *B*) hopping, and *η*_1_, *η*_2_ parametrize the spin-orbit coupling. We define *ε*_*AA*_(**k**) = *ε*_1D_(*k*_*x*_, *k*_*y*_, *k*_*z*_), *ε*_*BB*_(**k**) = *ε*_1D_(*k*_*y*_, *k*_*x*_, *k*_*z*_), and *ε*_*CC*_(**k**) = *ε*_2D_(*k*_*x*_, *k*_*y*_, *k*_*z*_), and we retain the following terms in the matrix elements:

5 $${\varepsilon }_{1{{{{{{{\rm{D}}}}}}}}}({k}_{\parallel },{k}_{\perp },{k}_{z})=\, - {\mu }_{1{{{{{{{\rm{D}}}}}}}}}-2{t}_{1}\cos ({k}_{\parallel })-2{t}_{2}\cos ({k}_{\perp })-4{t}_{3}\cos ({k}_{\parallel })\cos ({k}_{\perp })\\ - 8{t}_{4}\cos ({k}_{\parallel }/2)\cos ({k}_{\perp }/2)\cos ({k}_{z}/2)-2{t}_{5}\cos (2{k}_{\parallel })\\ - 4{t}_{6}\cos (2{k}_{\parallel })\cos ({k}_{\perp }) -2{t}_{7}\cos (3{k}_{\parallel}),$$

6 $${\varepsilon }_{2{{{{{{{\rm{D}}}}}}}}}({{{{{{{\bf{k}}}}}}}})=\, - {\mu }_{2{{{{{{{\rm{D}}}}}}}}}-2{\bar{t}}_{1}[\cos ({k}_{x})+\cos ({k}_{y})]-2{\bar{t}}_{2}[\cos (2{k}_{x})+\cos (2{k}_{y})]\\ - 4{\bar{t}}_{3}\cos ({k}_{x})\cos ({k}_{y}) -4{\bar{t}}_{4}[\cos (2{k}_{x})\cos ({k}_{y})+\cos (2{k}_{y})\cos ({k}_{x})]\\ - 4{\bar{t}}_{5}\cos (2{k}_{x})\cos (2{k}_{y}) -4{\bar{t}}_{6}[\cos (3{k}_{x})\cos ({k}_{y})+\cos (3{k}_{y})\cos ({k}_{x})]\\ - 2{\bar{t}}_{7}[\cos (3{k}_{x})+\cos (3{k}_{y})] - 8{\bar{t}}_{8}\cos ({k}_{z}/2)\cos ({k}_{x}/2)\cos ({k}_{y}/2),$$

7 $${\varepsilon }_{AB}({{{{{{{\bf{k}}}}}}}})=- 8\tilde{t}\sin ({k}_{x}/2)\sin ({k}_{y}/2)\cos ({k}_{z}/2).$$

Here the first Brillouin zone is defined as BZ = [−*π*, *π*]^2^ × [ − 2*π*, 2*π*]. For the four values of *c*-axis compression *ε*_*zz*_ = 0, − 0.0075, − 0.015, − 0.020 we extract the entire set of parameters from DFT calculations consistent with Fig. 1; see Table 1.

**Table 1:** **Tight-binding parameters**. These parameters (in units of meV) were used for Eqs. (4), (5), (6), and (7), yielding Fig. 5a

∣*ε*_*zz*_∣	*μ*_1D_	*t*_1_	*t*_2_	*t*_3_	*t*_4_	*t*_5_	*t*_6_	*t*_7_	*μ*_2D_	${\bar{t}}_{1}$	${\bar{t}}_{2}$	${\bar{t}}_{3}$	${\bar{t}}_{4}$	${\bar{t}}_{5}$	${\bar{t}}_{6}$	${\bar{t}}_{7}$	${\bar{t}}_{8}$	$\tilde{t}$	*η*_1_	*η*_2_
0.00	316	296	53	− 16	17	− 57	− 15	− 12	433	370	− 6	123	20	14	3	3	− 2	9	− 51	− 51
0.0075	294	284	54	− 16	18	− 56	− 15	− 10	437	369	− 5	122	20	14	3	3	− 3	10	− 51	− 51
0.015	273	271	55	− 17	19	− 55	− 15	− 9	441	368	− 5	121	20	13	3	3	− 3	10	− 51	− 51
0.020	259	264	56	− 18	20	− 55	− 15	− 8	443	367	− 4	120	20	13	3	3	− 3	11	− 52	− 51

For the interactions we use the (on-site) Hubbard–Kanamori Hamiltonian

8 $${H}_{I}=\, \frac{U}{2}\mathop{\sum}\limits_{i,a,s\ne {s}^{\prime}}{n}_{ias}{n}_{ia{s}^{\prime}}+\frac{{U}^{\prime}}{2}\mathop{\sum}\limits_{i,a\ne b,s,{s}^{\prime}}{n}_{ias}{n}_{ib{s}^{\prime}}\\+\frac{J}{2} \mathop{\sum}\limits_{i,a\ne b,s,{s}^{\prime}}{c}_{ias}^{{{{\dagger}}} }{c}_{ib{s}^{\prime}}^{{{{\dagger}}} }{c}_{ia{s}^{\prime}}^{{{{\dagger}}} }{c}_{ibs}^{{{{\dagger}}} }+\frac{{J}^{\prime}}{2}\mathop{\sum}\limits_{i,a\ne b,s\ne {s}^{\prime}}{c}_{ias}^{{{{\dagger}}} }{c}_{ia{s}^{\prime}}^{{{{\dagger}}} }{c}_{ib{s}^{\prime}}^{{{{\dagger}}} }{c}_{ibs}^{{{{\dagger}}} },$$

where *i* is site, *a* is orbital, and ${n}_{ias}={c}_{ias}^{{{{\dagger}}} }{c}_{ias}$ is the density operator. We further assume that ${U}^{\prime}=U-2J$ and ${J}^{\prime}=J$^52^. In the weak-coupling limit this leaves *J*/*U* as a single parameter fully characterizing the interactions.

**Fig. 9:**
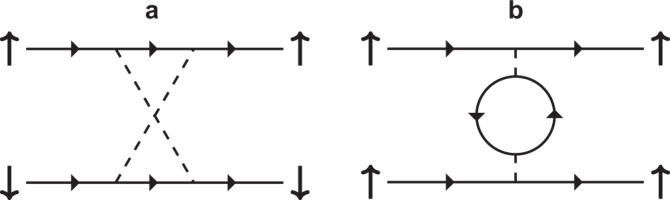
Two-particle interaction vertex diagrams. Second-order diagrams taken into account in Γ in **a** the even-parity channel, and **b** the odd-parity channel. The vertical arrows denote pseudo-spin, and the dashed lines contain all the terms of Eq. (8). The approach is asymptomatically exact in the weak-coupling limit, *U*/*t* → 0.

In the linearized gap equation (2) the (dimensionless) two-particle interaction vertex $\bar{{{\Gamma }}}$ is defined as^48^

9 $${\bar{\Gamma }}({{{{{\bf{k}}}}}}_{\mu },{{{{{\bf{k}}}}}}_{\nu })=\sqrt{\frac{{\rho }_{\mu }{\bar{v}}_{\mu }}{{v}_{\mu }({{{{{\bf{k}}}}}}_{\mu })}} {\Gamma }({{{{{\bf{k}}}}}}_{\mu },{{{{{\bf{k}}}}}}_{\nu })\sqrt{\frac{{\rho }_{\nu }{\bar{v}}_{\nu }}{{v}_{\nu }({{{{{\bf{k}}}}}}_{\nu })}},$$

where ${\rho }_{\mu }=\vert {S}_{\mu }|/[{\bar{v}}_{\mu }{(2\pi )}^{3}]$ is the density of states, and $1/{\bar{v}}_{\mu }={\int}_{{S}_{\mu }}{{{{{{{\rm{d}}}}}}}}{{{{{{{\bf{k}}}}}}}}/\left(|{S}_{\mu }|{v}_{\mu }({{{{{{{\bf{k}}}}}}}})\right)$. Here, Γ is the irreducible two-particle interaction vertex which to leading order retains the diagrams shown in Fig. 9.

An eigenfunction *φ* of Eq. (2) corresponding to a negative eigenvalue *λ* yields the superconducting order parameter

10 $${{\Delta }}({{{{{{{{\bf{k}}}}}}}}}_{\mu }) \sim \sqrt{\frac{{v}_{\mu }({{{{{{{{\bf{k}}}}}}}}}_{\mu })}{{\bar{v}}_{\mu }{\rho }_{\mu }}}\varphi ({{{{{{{{\bf{k}}}}}}}}}_{\mu }).$$

In the chosen pseudo-spin basis each eigenvector *φ* belongs to one of the ten irreducible representations of the crystal point group *D*_4*h*_^48,55^.

## Data Availability

The data that support the findings of this study are openly available from the Max Planck Digital Library^70^.
